# Supplementary material for: A highly dynamic F-actin network regulates transport and recycling of micronemes in Toxoplasma gondii vacuoles
Source: Nat Commun. 2019 Sep 13;10:4183. doi: 10.1038/s41467-019-12136-2 (PMC6744512; doi:10.1038/s41467-019-12136-2)
Supplement: Supplementary file 1 — Supplementary Information [file 41467_2019_12136_MOESM1_ESM.pdf]

Periz et al., A highly dynamic F-actin network regulates transport and recycling of micronemes in *Toxoplasma gondii* vacuoles

Supplementary Information

Supplementary Figure 1.

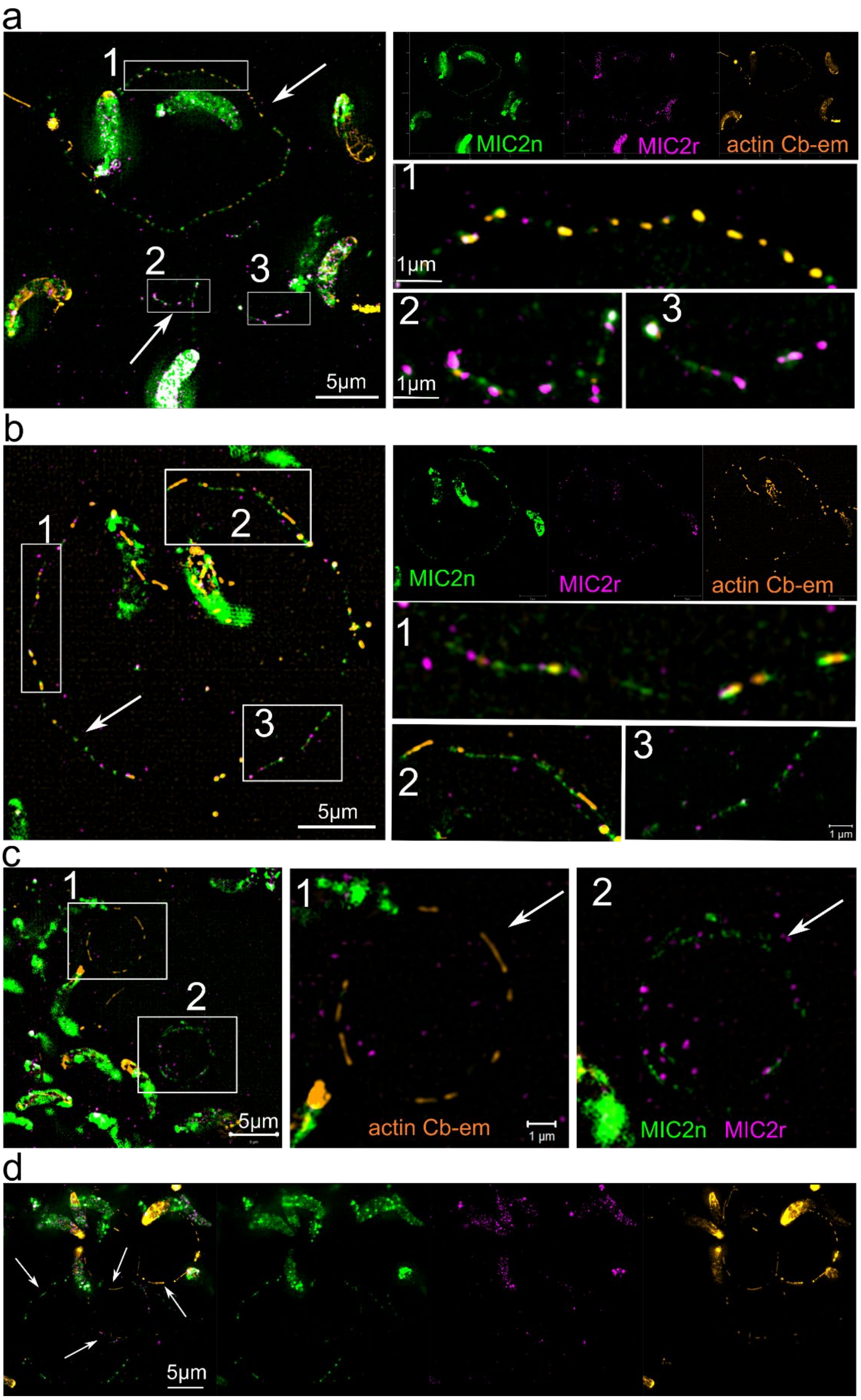

Supplementary Figure 1. Examples of single cell parasites showing gliding trail assays containing secreted MIC2 n (green), MIC2r (magenta) and actin (orange). a, 3D-SIM rendered image showing trails (white arrows); channels are shown independently (right top row); expanded views (1,2,3, white rectangles) show trails with MIC2n (green), MIC2r (magenta) and chromobody actin (orange). b,c,d, 2D SIM images show examples of parasite trails; c,d show actin trails not associated with MIC2 secretion. Parasitophorous vacuoles expressing MIC2 Halo and actin Cb-em PV were stained sequentially with TMR (MIC2r, magenta) and SiR (MIC2 n, green) in 20h interval. First dye added to 2-4 stages PV, and the second dye to 32 PVs (large vacuoles).PVs were left grow for further 18h. After that the parasites were purified using standard procedures(scratched, syringed and filtered) and allow to glide in foetal calf serum treated slides for 10 minutes. Scale bar 5 µm.

Supplementary Figure 2

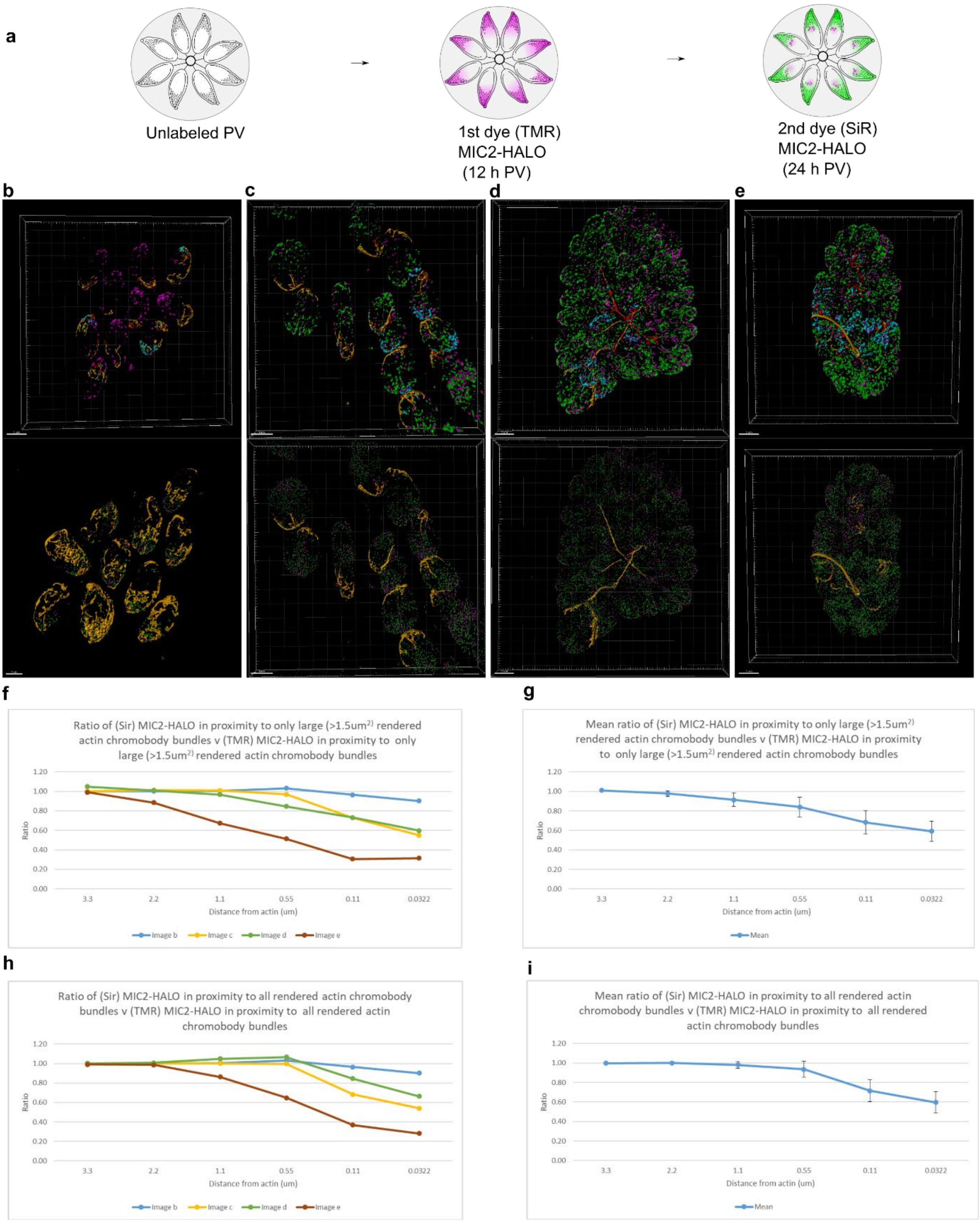

Supplementary Figure 2. Recycled and de novo MIC2 vesicle clusters associate with each other, and bridge actin bundles. **a**, 12h PVs expressing stably MIC2-HALO and actin-Cb-emerald (in orange) were sequentially labelled in a 12 h interval with Halo ligand coupled with TMR (in magenta) and Sir-rhodamine (in green) respectively and fixed after 36h. **b,c,d**,**e** Top panel, surface rendering of MIC2 vesicle populations. Total surface rendering of de novo (green), recycled (magenta), and actin bundle associated de novo (cyan) and recycled (red) vesicles. Bottom panel estimated number of total and associated vesicles to actin chromobody bundles. **f,** Quantification of the de novo/recycled (n/r) vesicle ratio in proximity to actin bundle in PV stages. Data correspond a,b,c,d panels . Estimated Number of vesicles and ratios of de novo (n) and recycled (r) are calculated at incremental distance to the actin bundle based on a voxel size with dimensions 0.0322 um (x,y dimension of a voxel), 1.1 um (the z dimension of a voxel). Measurements were taken at 0.55 um (5 voxels), 1.1 um (10 voxels), 2.2 um (20 voxels) and 3.3 um (30 voxels). Imaged was first adjusted the to have a gamma of 1.0 followed with an adjustment such that 90% of the pixels and the pixels which were in the lowest 10% of intensity values were removed (background). Threshold for rendering was then adjusted manually to ensure that every volume above background intensity was recognised by placing a rendered spot at the centre of the volume, or rendering a surface on the actin volume. Actin bundle is defined here as surface included large intact actin chromobody structures (>1,5um^2) chromobody bundle. **g** Mean of the de novo/recycled (n/r) vesicle ratio in proximity to actin bundle from PV stages in panels b,c,d,e. **h,** Quantification of the de novo/recycled (n/r) vesicle ratio in proximity to all F-actin detected with the chromobody in PV stages. Data correspond a,b,c,d panels. **i**, Mean of de novo/recycled (n/r) vesicle ratio in proximity to all F-actin detected with actin chromobody from PV stages in panels b,c,d,e. Scale bar 3μm, except b bottom panel, 2μm. Source data are provided as a Source Data file.

Supplementary Figure 3.

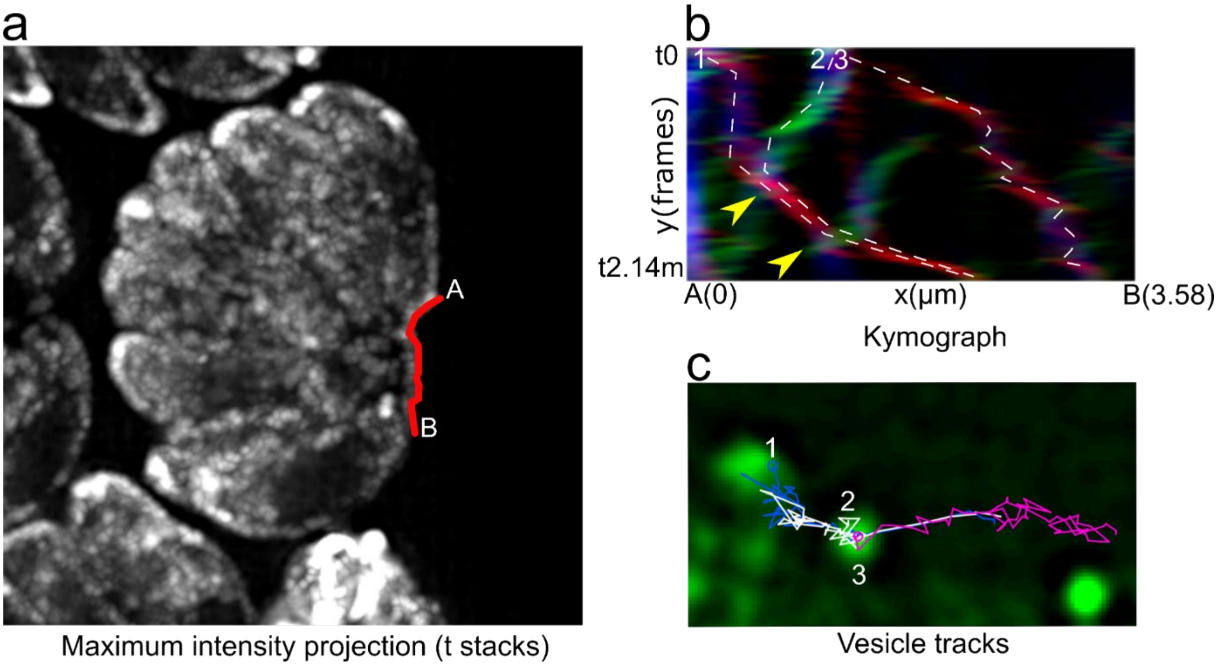

Supplementary Figure 3: Transport of MIC2 vesicles between parasites in PV. Kymograph analysis from supplementary video 2. a, Maximum intensity projection image. A-B line (red) describes the direction in which the kymograph is calculated. **b** Kymographs showing MIC2 vesicle flow of three particles. Tracks corresponding to vesicles 1,2 and 3 show static, anterograde, retrograde transport in blue, red and green. Flow tracks from vesicle 1 and 2 converged after approximately half of the recording time and move associated in an anterograde direction joining a third vesicle. Particle 3 dissociates from particle and undergoes anterograde movement. Movies were played at 25 frames per second. **c**, Manual particle tracking of vesicle 1,2 and 3 agrees with trajectories of three particles analysed in kymograph shown in b.

Supplementary Figure 4.

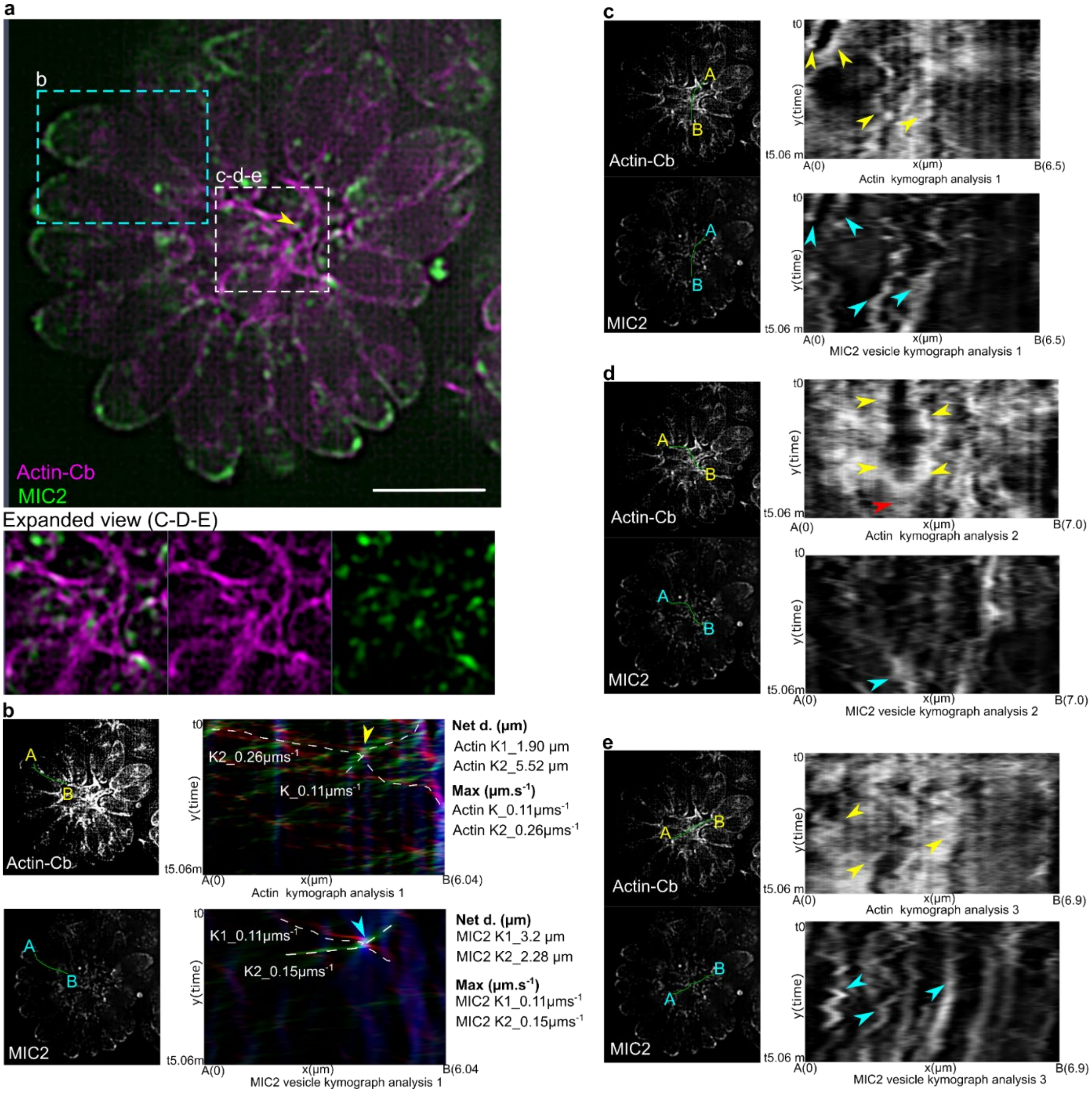

Supplementary Figure 4: Transport of MIC2 vesicles in the PV in cells and the residual body in the PV. a, SIM movie still showing MIC2 (green) and actin network (magenta). Expanded view. An actin network decorated with MIC2 vesicles (c,d,e) connecting cells inside the PV. **b**, Kymographs of actin and MIC2 vesicle in a cell. A-B line (green) describes the direction in which the kymograph is calculated. Top row. Tracks from an actin kymograph flow with different kinetics and directionality, including anterograde, retrograde and static flow. Bottom row. Broken line shows actin tracks with the same direction as MIC2 flow. **c,d,e**, Three independent kymographs measured in the residual body. **c**, Four tracks showing actin flow co-localised (yellow arrow heads) with MIC2 flow (cyan arrow heads). **d**, Two actin tracks (yellow arrow head) converged (red arrow head) and co-localised with accumulation of MIC2 flow. **e**, Three tracks showing mainly static actin flow co-localised (yellow arrow heads) with MIC2 flow (cyan arrow heads). Movies played at 12 frames per second. Scale bar 5μm. Source data are provided as a Source Data file.

Supplementary Figure 5.

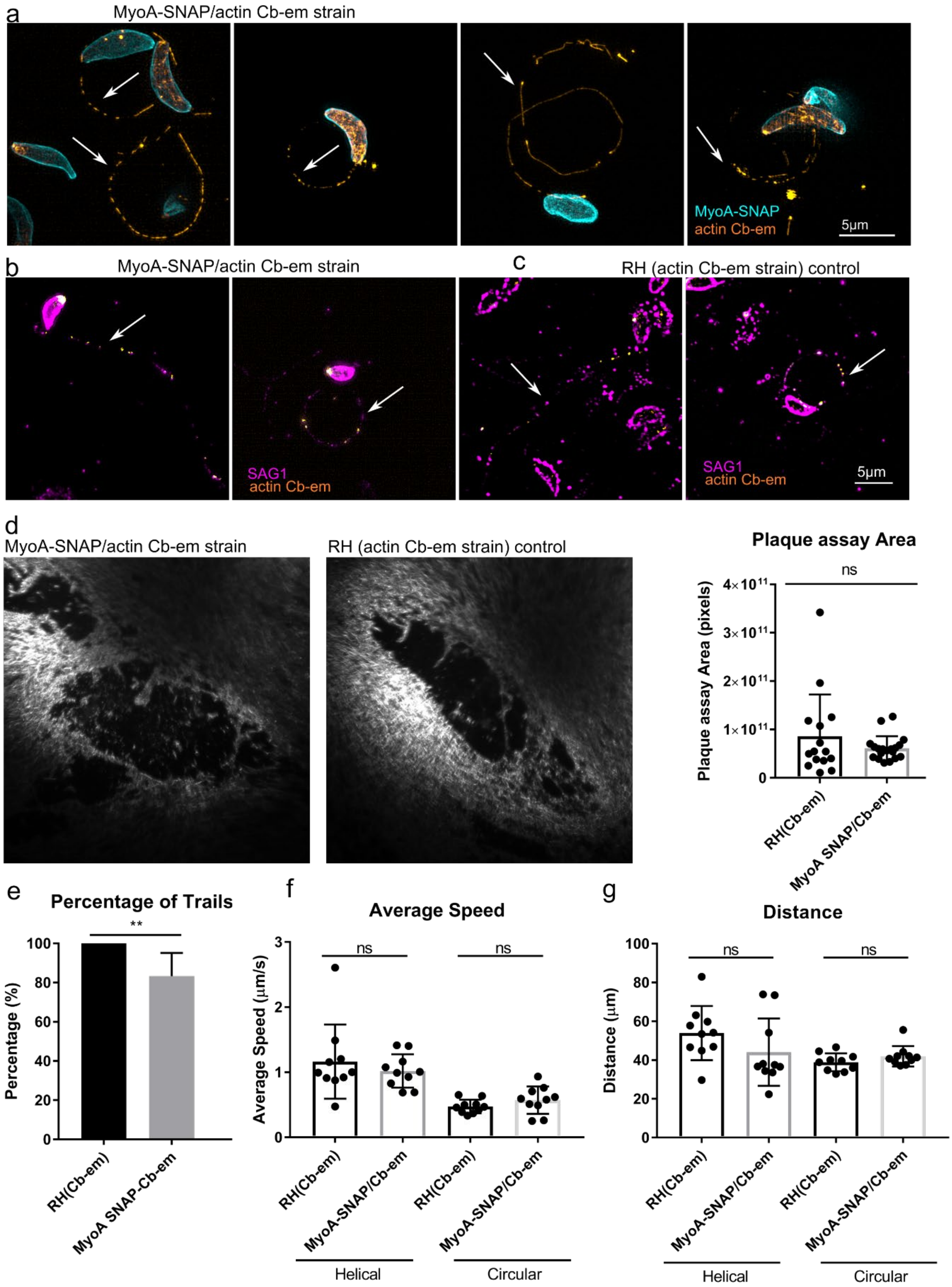

Supplementary Figure 5. Examples of trail assays in gliding parasites expressing MyoA-SNAP and actin Cb-em. Images show similar trailing behaviour in tagged parasites and controls. **a,** 3D-SIM rendered images of parasites expressing MyoA-SNAP/actin Cb-emerald. Images show representative trails (white arrows) containing actin chromobody (orange). MyoA-SNAP (cyan) was visualised with TMR ligand**. b, Trails of** MyoA-SNAP/actin Cb-emerald **parasites** showing trails labelled with of actin chromobody (orange) and surface membrane marker SAG1 (magenta) detected with a specific primary antibody and detected with a secondary antibody coupled to alexa 647 dye; Parasites were purified from parasitophorous vacuoles using standard procedures (scratched, syringed and filtered) and allow to glide in foetal calf serum treated slides for 30 minutes at 37°C. Scale bar 5 µm. **c** Examples of trails from control parasites (RH strain) expressing actin Cb-em; **d** Plaque assay Left. Representative image of plaque area size in in control and RH (Cb-emerald) and MyoA-SNAP/Cb-emerald. Right. Growth assay of indicated parasites. After 6 days growth no significant difference in growth rates can be observed. Quantification of plaque size in control and RH (Cb-emerald) and MyoA-SNAP/Cb-emerald (15 FOVs). **e,f,g** Trail assay comparing gliding rates between RH (Cb-emerald) and MyoA-SNAP/Cb-emerald. **e** Control parasites form slightly more trails (*$p<0.05$). (**f,g**) Comparison of gliding motility between control and MyoA-SNAP/Cb-emerald strain. Average speed and run length (**g,h**) are not significantly different for helical and circular motility. Parasites were tracked with ICY software. n = 10 individual events for each condition. All assays were conducted in triplicates. Datasets were compared using a Mann-withney test. Error bars represent SD from three independent, biological replicates. Error bars for e, f, g represent 95 % CI. *$p<0.05$. Scale bar 5µm Source data are provided as a Source Data file.

Supplementary Figure 6

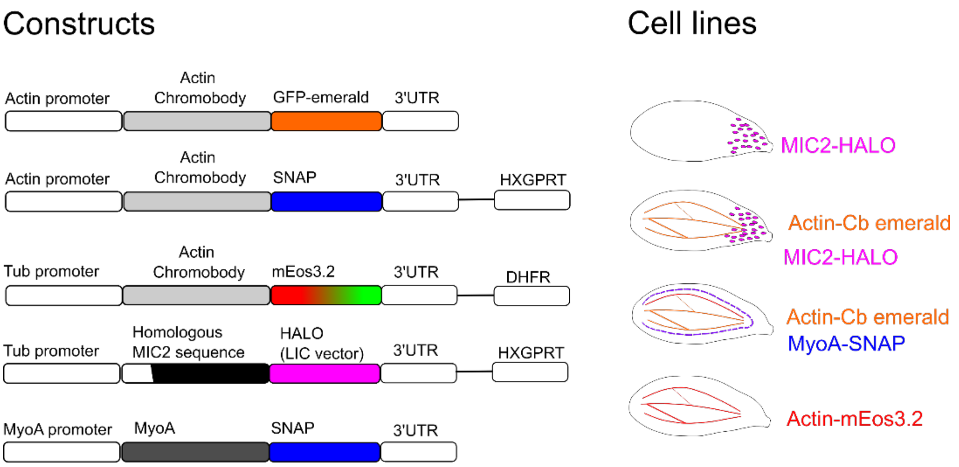

Supplementary Figure 6. Schematic of constructs and cell lines used in this work.

Dyes, primary antibodies

**Supplementary Table 1**			
Dye	Concentration	Stock	
TMR HALO	160 nM	5mM G8251	Promega
Oregon green HALO	160 nM	1 mM G280A	Promega
SiR HALO	160 nM	1mM RAH STFC	STFC
TMR SNAP	120 nM	0.6mM S9105S	NEB
Alexa 647 SNAP	120 nM	1mM S9136S	NEB
505* dye SNAP	120 nM	1mM S9103S	NEB
SiR-Tubulin	0.5 μM	1mM CY-SC002	Cytoskeleton Inc
α-IMC1(mouse)	1/1000 dilution	Professor Gary Ward	University of Vermont
α-mouse-Alexa 647	1/3000 dilution	A212327 2mg/ml	Life technology
α-mouse-SAG1	1/1000 dilution	Dr Sebastian Lourido	MIT
Jasplakinolide	100 nM	J4580-100	Sigma
Cytochalasin D	2 μM	C8273-1mg	Sigma
