# Supplementary material for: A highly dynamic F-actin network regulates transport and recycling of micronemes in Toxoplasma gondii vacuoles
Source: Nat Commun. 2019 Sep 13;10:4183. doi: 10.1038/s41467-019-12136-2 (PMC6744512; doi:10.1038/s41467-019-12136-2)
Supplement: Supplementary file 3 — Reporting Summary [file 41467_2019_12136_MOESM3_ESM.pdf]

nature research

Corresponding author(s): Javier Periz
Markus Meissner

Last updated by author(s): Aug 8, 2019

Reporting Summary

Nature Research wishes to improve the reproducibility of the work that we publish. This form provides structure for consistency and transparency in reporting. For further information on Nature Research policies, see Authors & Referees and the Editorial Policy Checklist.

Statistics

For all statistical analyses, confirm that the following items are present in the figure legend, table legend, main text, or Methods section.

n/a	Confirmed	
☐	☒	The exact sample size (*n*) for each experimental group/condition, given as a discrete number and unit of measurement
☐	☒	A statement on whether measurements were taken from distinct samples or whether the same sample was measured repeatedly
☐	☒	The statistical test(s) used AND whether they are one- or two-sided<br>*Only common tests should be described solely by name; describe more complex techniques in the Methods section.*
☐	☒	A description of all covariates tested
☒	☐	A description of any assumptions or corrections, such as tests of normality and adjustment for multiple comparisons
☐	☒	A full description of the statistical parameters including central tendency (e.g. means) or other basic estimates (e.g. regression coefficient) AND variation (e.g. standard deviation) or associated estimates of uncertainty (e.g. confidence intervals)
☐	☒	For null hypothesis testing, the test statistic (e.g. *F*, *t*, *r*) with confidence intervals, effect sizes, degrees of freedom and *P* value noted<br>*Give P values as exact values whenever suitable.*
☒	☐	For Bayesian analysis, information on the choice of priors and Markov chain Monte Carlo settings
☒	☐	For hierarchical and complex designs, identification of the appropriate level for tests and full reporting of outcomes
☒	☐	Estimates of effect sizes (e.g. Cohen's *d*, Pearson's *r*), indicating how they were calculated

Our web collection on statistics for biologists contains articles on many of the points above.

Software and code

Policy information about availability of computer code

Data collection	ImageJ FIJI 1.52h, Icy BioImage Analysis Unit 1.9.9.1 with Manual Tracking plugin Id ICY-K2V9O1, Zen 2 (Blue edition) 2.0.14283.302
Data analysis	Graphpad PRISM 7 ver 7.03, Imaris Imaging Software 6 and 9.2(Bitplane, Oxford Instruments), KymographTracker plugin from ICY,http://icy.bioimageanalysis.org/plugin/KymographTracker Colour code kymographs generated using KymographClear plugin on Image J ; SMLM code described in PMID: 27582387

For manuscripts utilizing custom algorithms or software that are central to the research but not yet described in published literature, software must be made available to editors/reviewers. We strongly encourage code deposition in a community repository (e.g. GitHub). See the Nature Research guidelines for submitting code & software for further information.

Data

Policy information about availability of data

All manuscripts must include a data availability statement. This statement should provide the following information, where applicable:

- Accession codes, unique identifiers, or web links for publicly available datasets
- A list of figures that have associated raw data
- A description of any restrictions on data availability

Data available on request from the authors

Field-specific reporting

Please select the one below that is the best fit for your research. If you are not sure, read the appropriate sections before making your selection.

☒ Life sciences ☐ Behavioural & social sciences ☐ Ecological, evolutionary & environmental sciences

For a reference copy of the document with all sections, see nature.com/documents/nr-reporting-summary-flat.pdf

Life sciences study design

All studies must disclose on these points even when the disclosure is negative.

Sample size	Sample size for microscopy imaging calculation not possible. A sample size include representative images of the replication vacuoles in multiple sizes one, two, four, eight and larger than 16 cell cells (large vacuoles).
Data exclusions	vacuole specimen in focus were selected for analysis
Replication	Representative images of MIC2 recycling were choosen from observations done at least three times across different days, with three complementary microscopy methods fixed SIM, SMLM, wide field fluorececence microcopy. Observation results were replicated by performiong with two different combinations of fluorescence ligand pairs (oregon greeen/TMR and TMR/SiR). These observations covered a wide range of parasite replication stages from single cell to large vacuoles. Observations of actin bundles were replicated with at least three complementary tags (chromobody emerald, chromobody-SNAP and chromobody-mEos3.2
Randomization	During image acquisition and manual counting, random fields of views were used by manually changing the stage location in the microscope. For live imaging capturing specific trafficking events were counted upon encountering as they the parasite vacuoles are not synchronysed.
Blinding	No blinding was possible due since each tagged strain presents a very specific phenotype that can be easily recognised by the person carrying out the assay.

Reporting for specific materials, systems and methods

We require information from authors about some types of materials, experimental systems and methods used in many studies. Here, indicate whether each material, system or method listed is relevant to your study. If you are not sure if a list item applies to your research, read the appropriate section before selecting a response.

Materials & experimental systems

n/a	Involved in the study	
☒	☐	Antibodies
☐	☒	Eukaryotic cell lines
☐	☒	Palaeontology
☒	☐	Animals and other organisms
☒	☐	Human research participants
☒	☐	Clinical data

Methods

n/a	Involved in the study	
☒	☐	ChIP-seq
☒	☐	Flow cytometry
☒	☐	MRI-based neuroimaging

Eukaryotic cell lines

Policy information about cell lines

Cell line source(s)	Toxoplasma gondii RH cell lines and all Toxoplasma gondii were generated based on this original cell line. The cell line is available in (ATCC® 50174D™). The MIC2 cell line, was generated based on this cell line after deletion of the deltaKU80 locus to allow integration of large genomic sequences into the parasite.<br>Human Foreskin Fibroblasts (HFF) were used as host cells and are commercially available (ATCC® SCRC-1041™).
Authentication	Toxoplasma gondii RH cell lines and all Toxoplasma gondii mutants were generated based on this original cell line. MIC2, Chromobody cell lines tested for PCR and phenotypic analysis . MyoA cell line was tested with a phenotypic analysis and derived from published strains PMID: 28100223.
Mycoplasma contamination	Mycoplasma tests were negative for the HFF. Toxoplasma gondii lines were not tested.
Commonly misidentified lines<br>(See ICLAC register)	None of our cell lines currently appear in the database

Palaeontology

Specimen provenance	na
Specimen deposition	na
Dating methods	na

☐ Tick this box to confirm that the raw and calibrated dates are available in the paper or in Supplementary Information.
