# Supplementary material for: A highly dynamic F-actin network regulates transport and recycling of micronemes in Toxoplasma gondii vacuoles
Source: Nat Commun. 2019 Sep 13;10:4183. doi: 10.1038/s41467-019-12136-2 (PMC6744512; doi:10.1038/s41467-019-12136-2)
Supplement: Supplementary file 4 — Description of Additional Supplementary Files [file 41467_2019_12136_MOESM4_ESM.pdf]

Description of Additional Supplementary Files

File Name: Supplementary Movie 1
Description: SIM movie of 2 stage PV showing association and co-localisation of recycled (magenta) and de novo synthesised (green) MIC2 vesicles. 25 frames per second. Scale bar 5µm

File Name: Supplementary Movie 2
Description: Left panel SIM movie showing recycling (green track) and lateral transport (blue track) of MIC2 vesicles (green). Green track shows recycling of MIC2 vesicle from the posterior end into a daughter cell. Microtubules are stained with Sir-tubulin (magenta). Movie played at 25 frames per second. Right panel, SIM movie showing exchange of three MIC2 vesicles (green) and arrows (blue, white and magenta) in the residual body of a 8 stage PV. Microtubules are stained with Sir-tubulin (magenta). Movie played at 25 frames per second. Scale bar 5µm.

File Name: Supplementary Movie 3
Description: Three SIM movies showing transport of MIC2 vesicles (green) on an F-actin network (orange) connecting the posterior ends of a 4 stage PV. Left movie actin filament support MIC2 association and transport (white arrow). Central movie F-actin in the RB associated with active MIC2 transport (white arrow). Right movie, a cytoplasmic F-actin network is associated with MIC2 in replicating cells (white arrow). Movie played at 12 frames per second. Scale bar 5µm

File Name: Supplementary Movie 4
Description: SIM movie showing transport of MIC2 vesicles (green) on a dynamic F-actin network (orange). A complex network in the residual body connect the parasites in the PV. Movie played at 25 frames per second. Scale bar 5µm.

File Name: Supplementary Movie 5
Description: SIM movie showing transport of MIC2 vesicles (green) on a mobile F-actin network (magenta) located in the residual body. A complex network in the residual body connect the parasites in the PV. Movie played at 25 frames per second. Scale bar 5µm.

File Name: Supplementary Movie 6
Description: Wide field fluorescence movie in a 4 stage PV shows multidirectional MIC2 vesicle (green) transport on an actin network (red), connecting parasites inside the PV. Scale bar 5µm.

File Name: Supplementary Movie 7
Description: 3D SIM reconstruction showing association of MIC2 vesicles (green) on a continuous F-actin network (orange) connecting daughter cells to the residual body. Actin Cb-SNAP (orange), MIC2-HALO (coupled with Oregon green, in green) 12 frames per second. Scale bar 5µm

File Name: Supplementary Movie 8
Description: (Left). 3D SIM reconstruction of an 8 cell PV showing association of MIC2- HALO(r) and (n) vesicles (in magenta and green respectively) with a continuous F-actin network (orange) that connects early stage daughter cells with the mother cell and to each other through a actin network in the residual body. (Right) F-actin network shown alone labelled with actin Cb-em. 12 frames per second. MIC2-HALO (r, in magenta) was labelled with TMR after 6h post invasion; MIC2-HALO (n) is labelled with SiR after 24h post invasion. Scale bar 5µm

File Name: Supplementary Movie 9

Description: SIM movie showing actin flow (orange) in cytoplasmic location interacting with Myosin A vesicles. Parasite periphery labelled with MyoA (cyan), Actin Cb-em (orange). b, SIM movie and expended views showing actin network (orange) supporting recycling and transport of MyoA vesicles (cyan). 12 frames per second. c, SIM movie showing transient association of actin bundles (orange) moving in a counter-clockwise direction in the residual body of a PV. MyoA (cyan). 25 frames per second. Scale bar 5µm

File Name: Supplementary Movie 10
Description: Transport and recycling of IMC material (MyoA-SNAP positive vesicles) is associated with F-actin. Top row SIM movie showing a four stage PV. F-Actin flow (orange) is associated with MyoA-SNAP positive vesicles (cyan colour, red arrow) in the residual body. Parasite periphery labelled with MyoA (cyan), Actin Cb-em (orange). Central row, PV treated with 200 nM JAS for 1h at 37°C. F-actin (orange) associates with MyoA-SNAP positive vesicles (cyan) in the RB. Bottom row, PVs treated with 2 µM CD for 1h at 37°C. MyoA-SNAP positive vesicles (cyan) are not found in the RB. After treatment with CD, F-actin network (orange) disappears, and the transport and recycling of MyoA-SNAP positive vesicles (cyan) in the RB is abrogated. 12 frames per second. Actin chromobody Cb-em was tagged with emerald GFP protein; Myo-SNAP is coupled with TMR ligand. One experiment triplicate observations of the events. Scale bar 5µm.

File Name: Supplementary Movie 11
Description: SIM movie showing F-actin flow and a mobile network of actin bundles that self-associate and connect parasites in the PV. Transient associations of mobile actin bundles (orange) in the PV. MyoA (cyan). Movie 25 frames per second. Scale bar 5µm.
